# Revolutionizing cancer immunotherapy: The dual role of clofazimine in enhancing efficacy and reducing toxicity

**DOI:** 10.1002/ctm2.1756

**Published:** 2024-07-04

**Authors:** Yiran Zhu, Yong Lu

**Affiliations:** ^1^ Houston Methodist Cancer Center/Weill Cornell Medicine Houston Texas USA

**Keywords:** cancer immunotherapy, clofazimine, immune‐related adverse events, immune checkpoint blockade

## 1 INTRODUCTION

The emergence of immune checkpoint blockade (ICB) as a cornerstone of cancer immunotherapy has revolutionized oncology by harnessing the body's immune system to combat malignancies. ICBs' effectiveness is curtailed by limited response rates and the prevalence of immune‐related adverse events (irAEs). This commentary discusses ground‐breaking research by Xue et al.,^1^ highlighting the significant potential of Clofazimine (CLF) in overcoming these hurdles.

## 2 THE CHALLENGES OF CURRENT ICB THERAPIES

**FIGURE 1:**
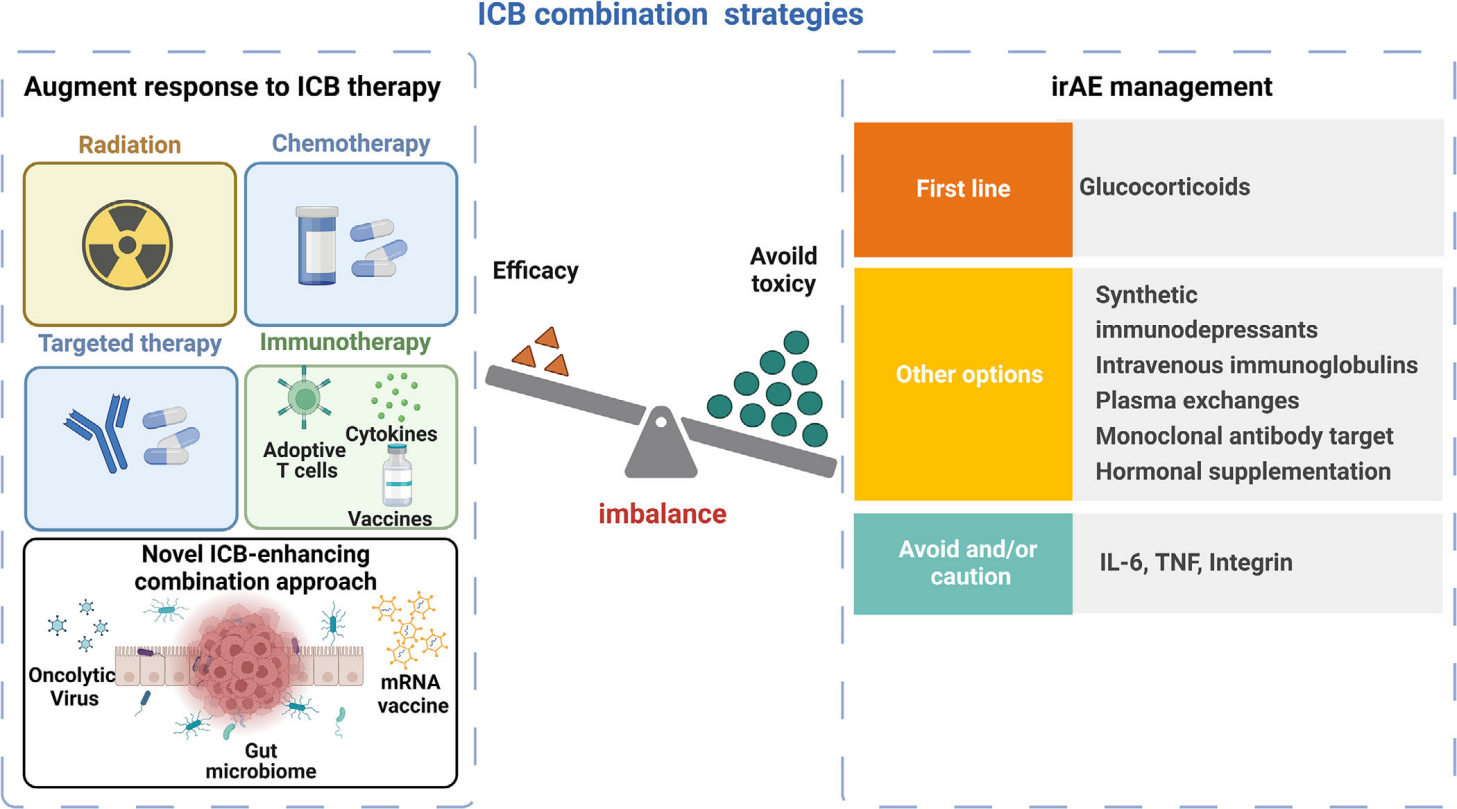
Overview of combination strategies with ICB therapy. The combination strategies with immune checkpoint blockade (ICB) therapy evolved from conventional treatments (such as chemotherapy, targeted therapy, and radiotherapy) toward innovative methodologies that integrate ICB to counter resistance. The therapeutic strategies for managing immune‐related adverse events (irAEs), prioritizing glucocorticoids unless endocrine issues arise, are also listed. However, there is the ongoing challenge of balancing the efficacy of ICB with the mitigation of irAEs.

The advent of ICB therapy, primarily through the combination of anti‐cytotoxic T‐lymphocyte associated protein 4 (anti‐CTLA‐4) and anti‐PD‐1/L1, has led to notable improvements in response rates and overall survival and offered hope for patients resistant to anti‐PD‐1/L1 monotherapy. However, resistance to ICB and the development of irAEs pose significant challenges. Here, we briefly review the contemporary strategies to augment response to ICB therapy and methods to mitigate associated irAEs (Figure 1).

The efficacy and resistance to ICB therapy are influenced by two main factors: (1) intrinsic elements such as tumour genetics, tumour microenvironment and immune regulation (e.g. systemic factors and microbiota), and (2) extrinsic factors such as environmental determinants. Several combination strategies have been explored to overcome resistance, from integrating conventional therapies (such as chemotherapy, targeted therapy, radiotherapy, and immunotherapy) with ICB to innovative approaches that pair oncolytic viruses and vaccines with ICB.^2^ A few of these combination therapies have received Food and Drug Administration (FDA) approval.^3^ However, these combination therapies will likely be associated with an enhanced prevalence of irAEs, emphasizing the importance of balancing efficacy with reduced toxicity.

irAEs can affect almost any organ, varying from mild to life‐threatening. Over two‐thirds of cancer immunotherapy‐related irAEs are attributed to ICB, especially when CTLA‐4 and PD‐1 inhibitors are combined, leading to significantly higher toxicity that is additive rather than synergistic.^4^ Management of irAEs primarily involves glucocorticoids for non‐endocrine irAEs and other therapies like intravenous immunoglobulins for neurological and haematological irAEs. For steroid‐refractory or persistent cases, monoclonal antibodies such as Infliximab, Tocilizumab and Natalizumab may offer relief.^5^ However, these treatments could potentially diminish the effects of ICB and come with their own set of adverse effects and infection risks, which limits their use in some irAE scenarios. Therefore, identifying combination strategies that enhance therapeutic response while minimizing irAE risks is crucial for maximizing ICB therapies' benefits.

## 3 CLF CONCURRENTLY BOLSTERS DUAL ICB EFFICACY AND AMELIORATES IRAES

**FIGURE 2:**
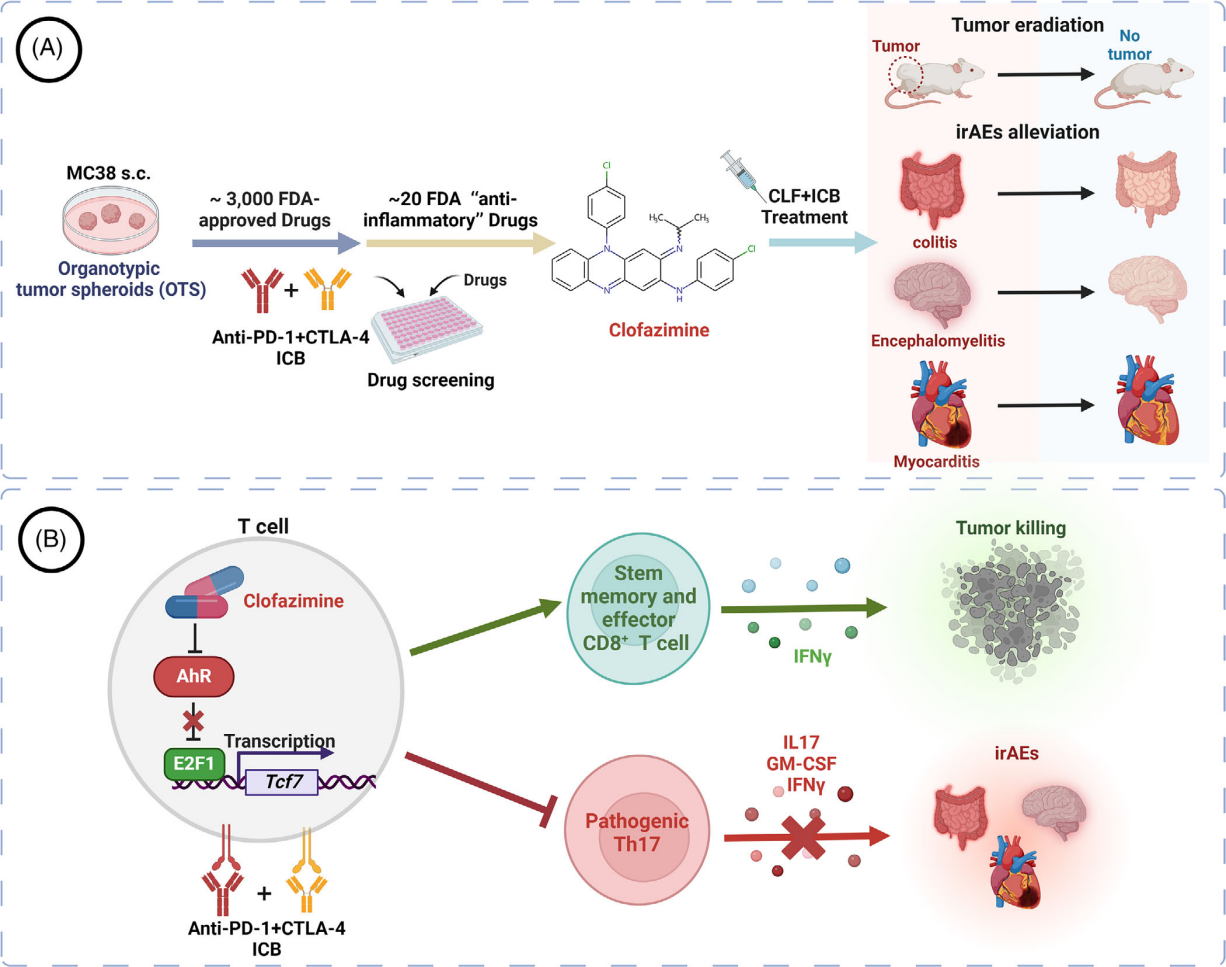
Clofazimine's dual role in enhancing efficacy and mitigating toxicity. (A) Research overview: Our study utilized organotypic tumor spheroids (OTS) for two rounds of drug screening, through which clofazimine (CLF) emerged as a promising agent capable of augmenting efficacy while diminishing toxicity in conjunction with immune checkpoint blockade (ICB). Animal studies further validated the combined treatment of CLF and ICB, showcasing its effectiveness in tumor eradication and mitigation of immune‐related adverse events (irAEs). (B) Mechanism of Action: CLF operates as an AhR inhibitor, blocking E2F1‐mediated *Tcf7* transcription. Consequently, CLF ensures an enhanced cytotoxic T lymphocyte response within the context of ICB treatment while simultaneously neutralizing pathogenic Th17 cells, effectively preventing irAEs.

In our recent studies, we embarked on a mission to address the dual challenges of irAEs and the resistance to dual ICB commonly observed in cancer therapies (Figure 2A). Utilizing a comprehensive approach, they screened an extensive library of more than 3000 FDA‐approved drugs using the MC38 organotypic tumour spheroids (OTSs) model. Their efforts led to identifying CLF as a promising agent capable of overcoming resistance to ICB, particularly notable since CLF falls within the “anti‐inflammatory/immunomodulatory” drugs, showcasing its potential in mitigating irAEs effectively. Delving deeper, we carried out a comprehensive series of experiments using both murine‐derived and patient‐derived OTS models, as well as various mouse models, including MC38, D4M.3A, A20, and LL/2, alongside melanoma models. The incorporation of CLF alongside anti‐PD‐1+CTLA‐4 ICB treatment demonstrated a significant enhancement in tumour cell eradication. This combination therapy not only managed to clear advanced tumours but also led to tumour‐free survival across several models, a benchmark that neither ICB nor CLF alone could achieve. These findings illuminate an unprecedented role of CLF in counteracting ICB resistance across a spectrum of tumour models.

Focusing on the complications associated with ICB therapy, namely, severe irAEs such as colitis, neurological and cardiac toxicities, the study further explored the therapeutic potential of CLF by employing a diverse range of models. Remarkably, CLF significantly alleviated these conditions, outperforming conventional treatments like dose adjustments and steroids. This efficacy underscores the utility of CLF in enhancing the anti‐tumour response and reducing the incidence and severity of high‐grade irAEs, thereby offering a dual advantage in the context of cancer immunotherapy. Through additional validation in humanized NSG mice models, the study extended its implications to suggest a viable and effective strategy for optimizing ICB therapy outcomes.

## 4 MECHANISM OF ACTION OF CLF IN ICB THERAPY

CLF, renowned for its antimicrobial efficacy against Mycobacterium leprae and Mycobacterium tuberculosis, exhibits a broader spectrum of action, including notable anti‐tumor properties. These effects encompass targeting cancer cells directly and overcoming chemoresistance, partially by modulating pathways like Wnt and PPAR‐γ.^6^ However, the clinical application of CLF in cancer has been constrained by an incomplete understanding of its action mechanism, necessitating more research. Additionally, its anti‐inflammatory qualities imply interactions with immune system components that might affect anticancer efficacy.

Utilizing advanced methodologies such as single‐cell RNA sequencing, we demonstrated that combining ICB and CLF enhances memory gene signatures within CD8^+^ T cells, signalling a strengthened anti‐tumour response. Additionally, ATAC‐seq and gene array analyses have further uncovered that CLF amplifies the anti‐tumour capacity of tumour‐specific CD8^+^ T cells through the upregulation of E2F1‐induced *Tcf7* promoter activation. Moreover, prior research indicates that TCF‐1 (encoded by *Tcf7*) expression diverts CD4^+^ T cells from differentiating into pathogenic Th17 (pTh17) cells,^7^ of which are known to provoke irAEs in patients.^8^ We thus have corroborated that activating the E2F1‐TCF1 pathway diminishes the presence of pathogenic Th17 cells, effectively mitigating irAEs (Figure 2B).

## 5 CLINICAL TRANSLATION AND FUTURE PERSPECTIVES

In our studies, we utilized advanced methods such as drug screening, scRNA‐seq, and animal models to highlight CLF's potential in enhancing anti‐CTLA‐4 and anti‐PD‐1 therapies. These findings suggest that incorporating CLF into ICB treatments could improve response rates, extend survival, and minimize adverse effects, enhancing patient quality of life. Given the complexity of solid tumours and the heterogeneity of tumour responses in patients, it is paramount to refine drug delivery mechanisms and identify patient cohorts that would derive maximal benefit from this combinational therapy.^9^ Moreover, exploring CLF's compatibility with different ICB therapies could broaden effective cancer treatment options, marking a step toward optimizing patient outcomes and advancing personalized cancer care.

## AUTHOR CONTRIBUTIONS

*Concepualization*: Yong Lu and Yiran Zhu. *Writing*: Yiran Zhu. *Supervision and Funding acquisition*: Yong Lu.

## CONFLICT OF INTEREST STATEMENT

The authors declare no conflict of interest.

## ETHICS STATEMENT

The manuscript does not involve any research with human or animal subjects; ethical approval is not applicable to this manuscript.
